# Adaptive evolution of seed oil content in angiosperms: accounting for the global patterns of seed oils

**DOI:** 10.1186/s12862-016-0752-7

**Published:** 2016-09-09

**Authors:** Anushree Sanyal, Guillaume Decocq

**Affiliations:** 1 Unité “Ecologie et Dynamique des Systèmes Anthropisés (EDYSAN, FRE 3498 CNRS), Université de Picardie Jules Verne, 1 rue des Louvels, Amiens Cedex, FR-80037 France; 2 Institute for Organismal Biology, Systematic Biology, Uppsala University, Uppsala, 75236 Sweden

## Abstract

**Background:**

Studies of the biogeographic distribution of seed oil content in plants are fundamental to understanding the mechanisms of adaptive evolution in plants as seed oil is the primary energy source needed for germination and establishment of plants. However, seed oil content as an adaptive trait in plants is poorly understood. Here, we examine the adaptive nature of seed oil content in 168 angiosperm families occurring in different biomes across the world. We also explore the role of multiple seed traits like seed oil content and composition in plant adaptation in a phylogenetic and nonphylogenetic context.

**Result:**

It was observed that the seed oil content in tropical plants (28.4 %) was significantly higher than the temperate plants (24.6 %). A significant relationship between oil content and latitude was observed in three families Papaveraceae, Sapindaceae and Sapotaceae indicating that selective forces correlated with latitude influence seed oil content. Evaluation of the response of seed oil content and composition to latitude and the correlation between seed oil content and composition showed that multiple seed traits, seed oil content and composition contribute towards plant adaptation. Investigation of the presence or absence of phylogenetic signals across 168 angiosperm families in 62 clades revealed that members of seven clades evolved to have high or low seed oil content independently as they did not share a common evolutionary path.

**Conclusion:**

The study provides us an insight into the biogeographical distribution and the adaptive role of seed oil content in plants. The study indicates that multiple seed traits like seed oil content and the fatty acid composition of the seed oils determine the fitness of the plants and validate the adaptive hypothesis that seed oil quantity and quality are crucial to plant adaptation.

**Electronic supplementary material:**

The online version of this article (doi:10.1186/s12862-016-0752-7) contains supplementary material, which is available to authorized users.

## Background

Seed traits are crucial fitness-related traits that are expected to underpin survival and reproductive success of plants in different environments. Seed oils serve as the primary energy source to the developing embryo during the heterotrophic stage [1], prior to the initiation of photosynthesis. The quantity and quality of stored oils in seeds is crucial in determining plant fitness, germination success [2], emergence and establishment of a plant [3], especially annuals as they need to establish and reproduce successfully in the year they germinate and are therefore under selection [2]. Earliness of germination was positively correlated with seed lipid content and the seed area to mass ratio [4]. Thus, seed traits like seed size, weight and endosperm content determines to a great extent the reproductive success of plants. Studying seed oil content (total content and quality), a trait which is predicted to be under natural selection as it influences initial rapid growth will help us understand the evolutionary significance of seed oil content and will also be of great interest from an economical point of view as it will help in the breeding and cultivation of oilseed crops.

A relationship between oil content and biomes (temperate, subtropical and tropical), was predicted which suggested that greater packing of species and competitive interactions among tropical plants may select for a greater energy reserve in seeds [5]. The only study on the evolutionary pressures acting on seed oil content has explored the relationship between seed oil content and species habit and seed oil content and biomes in species from several families occurring across biomes [6]. The study showed that the seed oil content in plants increased with woodiness and shade tolerance. These patterns have been attributed to reproductive strategies (*r* or *K*) selection of the habit group. As the seed oil content in herbs, shrubs and trees increases progressively, concomitantly they devote a smaller portion of the resources to reproduction resulting in more *K-*selected forms. In the tropics, a greater prevalence of *K*-selected forms have been observed, where seeds rich in reserves were selected [5, 6]. However, trees have both *r* and *K*- selected forms. The perennials with high longevity can produce a large quantity of seeds and also those trees that live in the understory during the first part of their life history must accumulate more reserve lipids for rapid and steady initial growth, hence selection will act on seeds with more oil content. However, no significant differences in seed oil content was observed between vines, shrubs and shrubby trees of flora of several families across biomes. Furthermore, no significant difference was found between the oil content of plants of the same habit in the temperate, tropical and subtropical regions; with the exception of the herbs from the subtropical region which had seeds with greater oil content than the herbs from the other two regions.

In Levin’s [6] study no significant relationship was observed between seed oil content and latitude across families. Given sufficient genetic variation for seed oil content, selection at the microevolutionary level should produce predictable patterns [2]. Lack of genetic variation is some species within a family and within a clade might not reveal the pattern or there could be selection on other competing traits, and this lack of variation may prevent them from expanding their latitudinal or altitudinal range [2]. Studying the relationship between seed oil content and latitude will help us understand the role of seed oil content in plant adaptation.

**Fig. 1:**
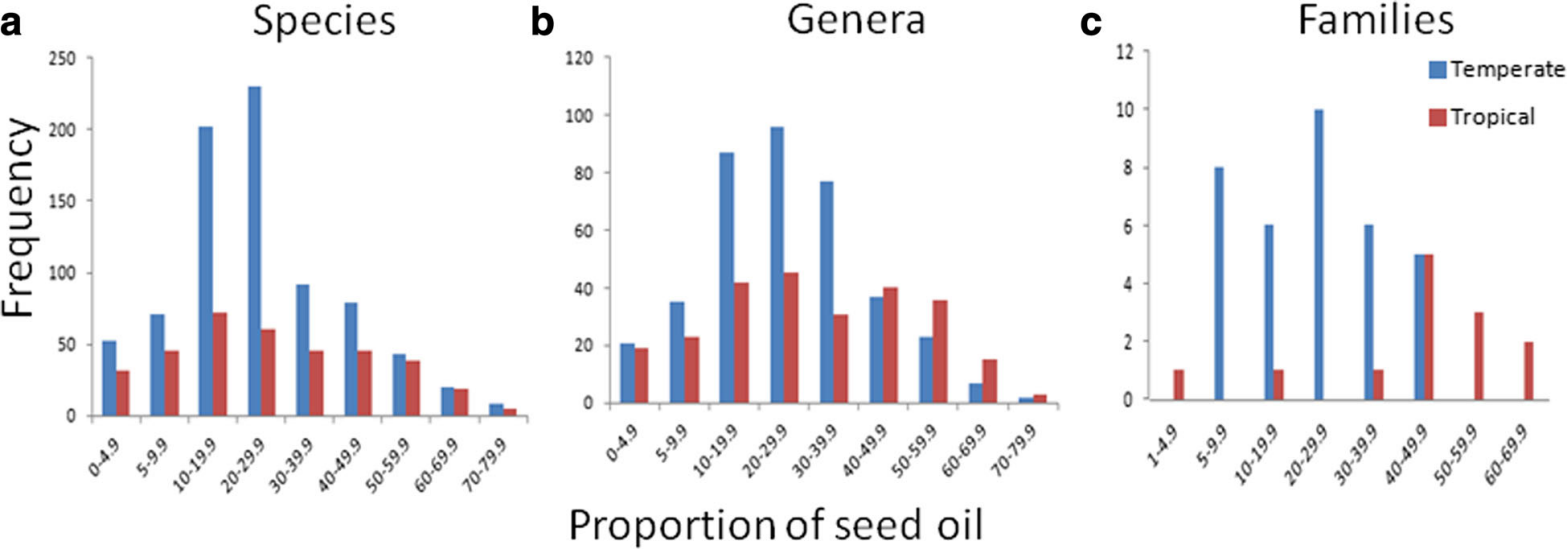
**a** Seed oil content distribution in strictly temperate and tropical families (**b**) Seed oil content distribution in strictly temperate and strictly tropical species across all families (**c**) Seed oil content distribution in strictly temperate and tropical genera across all families

Seed oils primarily accumulate neutral oils [7] which are composed of triacyglycerols (TAGs). Storage of energy rich TAG likely determines the fitness of most plants as it is an immediate energy source for seed germination and plant establishment for 80 % of the plants which rely on TAGs in their seeds [8] while fewer plant species rely on starches and proteins. The oil content can vary from 1 % in *Musa paradisiaca* to 76 % in *Chrysobalanus icaco* [9] (Fig. 1). The oil content varies within and among genera of the same families, among families [6] and within a single species [10]. However, despite the variation, seed oil content is under strict genetic control. The heritability of oil content typically exceeds 50 % [11, 12], and plant density, climate and mineral levels in the soil have little effect on the seed oil content [13–15]. The genetic variation and genetic control of seed oil content shows that seed oil content is under selection and subject to evolutionary change [6].

Several species of the Orchidaceae family have tiny seeds lacking endosperm [16] with scarce seed reserves, though the oil content can be as high as 32 % [17]. Epiphytic orchids produce very small seeds to colonize the canopy of the forests [18], need light during germination, depend on mycorrhizal fungi for the initial growth and there is evidence of the inability of the embryo to use its oil reserves (oil droplets inside embryo cells) in absence of an external source of simple sugars [19]. The disappearance of the cotyledons and the endosperm seems to have led to the loss of several biochemical capabilities of orchid seeds [20], including the ability to catabolize oils. In such cases it is likely that the seed oil will not be subjected to selection, but other seed characteristics, like the size and architecture will be under selection.

Previous studies have shown that seed oil content is regulated by *GLABRA2* (*GL2*), *TAG1*, *SUGAR-DEPENDENT1* (*SDP1*)*, PICKLE* (*PKL*), *HAIKU2* (*IKU2*) genes [21–28] and several transcription factors viz. *WRINKLED1* (*WRI1*), *LEAFY COTYLEDON1*(*LEC1*), *LEC2* [29–32]. In addition, *ABSCISSIC ACID INSENSITIVE 3* (*ABI3*), *FUSCA3* (*FUS3*) [33], soybean *DNA BINDING WITH ONE FINGER4* (*DOF4*) and *DOF11* have shown significant effects on seed oil levels [34]. Furthermore, the yeast *SLC1-1* gene can enhance seed lysophosphatidic acid acyltransferase activity and alter the oil content and composition of lipids [35]. The yeast cytosolic glycerol-3-phosphate dehydrogenase (*GPD1*) when expressed in transgenic oil-seed rape led to an increase in the level of glycerol-3- phosphate in developing seeds, and a 40 % increase in the final lipid content of the seed, with the protein content remaining substantially unchanged [36]. Studies have shown that the supply of fatty acids (FAs) is a limiting factor for oil accumulation in developing embryos [30]. Acetyl CoA carboxylase (*ACCase*), the first enzyme in FA synthesis, is considered to be a control point for carbon flux into FA synthesis [37]. Expression of *Arabidopsis* cytosolic *ACCase* in *B. napus* resulted in a 5 % increase in seed oil content [38]. A study has also shown the evolutionary pattern of the *FAE1* gene and its correlation with the FA erucic acid in seed oils in Brassicaceae [39]. Hence, evaluating the evolutionary pattern of these seed oil content genes in species from several families across the world will reveal consistent evolutionary patterns and will help us understand seed oil evolution in plants. In addition, detection of variations in the genes and correlation analysis between the genotype and phenotype and the presence or absence of phylogenetic signal on oil content will also reveal the adaptive nature of seed oil content.

Previous studies have investigated the variation in the patterns of seed oil composition (FA composition in seed oils) with latitude within and between species [2, 40]. These studies have shown that the proportions of saturated and unsaturated FAs and subsequently their melting points vary with latitude [2, 40]. On a per carbon basis, unsaturated FAs cost more to produce and yield less energy when oxidized than saturated FAs [41]. Thus, at lower latitudes, seeds with higher proportions of saturated oils would be favoured because they would have more energy for growth without delaying or slowing germination. At higher latitude and cooler germination temperatures, seeds that have a higher proportion of unsaturated oils may germinate earlier and/or more rapidly than seeds that are higher in saturated FAs. The extra potential energy in seeds with higher proportions of saturated oils would be wasted as evidence suggests that lipases that catalyze the removal of FAs from glycerol prior to *ß*-oxidation operate more rapidly on liquid substrates [42–44].

Linder’s [2] study showed that in *Helianthus annuus*, germination temperature selects for greater proportions of unsaturated FAs at higher latitude and releases energy faster at lower temperatures (like at higher latitudes). On the contrary, there is selection for greater proportions of saturated fatty acids at lower latitudes as saturated FAs store more energy per carbon atom. In Linder’s [2] study, the hypothesis was tested both in phylogenetic and non-phylogenetic contexts and the validity of the hypothesis was revealed at micro and macroevolutionary scales. In another study, the oilseed species, *Plukenetia volubilis*, had significantly higher proportions of unsaturated FAs at higher altitudes [45], which would be predicted as temperatures decrease at higher altitudes.

Studies analyzing the total content of seed oil of species in a phylogenetic and non-phylogenetic context are needed in order to elucidate the evolutionary trends of seed oil content on the assumption that selection has erased the effects of phylogenetic history. Levin’s [6] study on the relationship between seed oil content and latitude in a non-phylogenetic context failed to reveal any significant relationship. Here, we explore the differences in seed oil content in different biogegraphical regions and the relationships between seed oil content and latitude on species from several families representing flora across biomes in a phylogenetic and non-phylogenetic context to understand the adaptive role of seed oils in plant adaptation. The study also explored the importance of considering combinations of multiple genetically based traits (seed oil content and seed oil composition) along a latitudinal and climatic gradient as adaptive strategies.

## Methods

### Seed oil content and seed oil composition

Published seed oil content data for 2567 species of 168 families and seed oil composition of Sapindaceae, Sapotaceae and Papaveraceae families were obtained primarily from the Seed Oil Fatty Acids database (http://sofa.mri.bund.de/) [9] and from the literature [46]. The mean seed oil content and fatty acid proportions of all the available samples of a given species was calculated (Additional file 1: Table S1).

### Plant distribution

The data of the geographical distribution of 2567 species into tropical, temperate or subtropical regions was obtained from the information available in the Germplasm Resources Information Network (http://www.ars-grin.gov/) from the USDA, the Euro + Med PlantBase (http://www.emplantbase.org/home.html); the Plants For A Future database (http://www.pfaf.org/user/default.aspx), from literature [47] and from published works of local and regional flora [48]. In addition, the latitude information of the 2567 species was extracted at a global scale from the Global Biodiversity Information Facility (GBIF) (http://www.gbif.org/) by using the ‘rgbif’package [49] in the R programming software [50] (Additional file 1: Table S1).

### Statistical analysis

#### Testing whether seed oil content varies among biogeographical regions

The research hypothesis we tested was that there will be no change in the seed oil content in different biogeographical regions i.e. the temperate and the tropical regions. An unpaired *t*-test comparing the seed oil content in all tropical and temperate species and also the seed oil content between strictly tropical and strictly temperate species were conducted. Two-way ANOVA’s were performed for genera and families that had both tropical and temperate members, with latitude (tropical, temperate) and taxon (either family or genus) as main effects. One- way ANOVA was performed to test for differences between temperate and tropical members of families and genera with representative members from both temperate and tropical regions. The seed oil content was the dependent variable and latitude (temperate and tropical) was the independent variable. Furthermore, nested ANOVA with genera or families nested within latitude for genera and families which had only temperate or tropical members was performed.

#### Analysis of change in seed oil content proportions with latitude across and within families

The hypothesis tested was that there will be no change in the seed oil content with latitude in species from 168 families. An ordinary least-square (OLS) regression at the species, family and genera level were performed. The OLS regressions were performed with species’ mid-latitudinal range as the explanatory variable and species’ seed oil content as the response variable. Because the mid-latitudinal range position of a species is constrained between the poles and depends on its latitudinal range, it is likely that the relationship between species’ mid-latitudinal range and species’ seed oil content is affected by species’ latitudinal range. Hence, we included species’ latitudinal range as a covariate in all our models. Mid-latitudinal range position was computed using the following formula: minimum latitude + species’ latitudinal range/2), where species latitudinal range is the maximum latitude minus the minimum latitude. Furthermore, OLS regression analysis was performed on four families (Sapindaceae, Sapotaceae, Papaveraceae and Solanaceae) where the sample numbers were greater than 30.

#### Analysis of correlations between seed oil content and seed oil composition in sapindaceae, sapotaceae and papaveraceae

The hypothesis tested was that there will be no correlation between seed oil content and seed oil composition in three families Sapindaceae, Sapotaceae and Papaveraceae. The correlation analysis of seed oil content and seed oil composition will reveal the relationships between the different fatty acids and seed oil content and the effect of these relationships on the final proportions of each component and the role of multiple seed traits in adaptive evolution of plants. In addition, correlation between seed oil content and seed size was investigated in Papaveraceae. Pearson’s correlation coefficient (*r*) was performed for pairwise comparisons of oil content and the constituent fatty acids in the three families.

#### Testing for phylogenetic signal in the seed oil content across angiosperm families

The published angiosperm phylogeny (version 13) by the Angiosperm Phylogeny Group [51] was used to test for phylogenetic signals in seed oil content across angiosperms. The method used in Linder’s [2]) study was followed to test for phylogenetic signals. The mean seed oil content of all the species of each family was mapped onto the phylogeny, the tree was then pruned of families for which seed oil content data was lacking. The pruned tree had 2571 terminal taxa and consisted of 2519 species, 965 genera and 168 families. There were 13 tropical clades and 34 temperate clades at the family level (Additional file 1: Table S1). Since, tropical clades were fewer than the temperate clades, tropical clades with at least two species were paired with temperate clades with a minimum of two species for which seed oil content were known [2]. Clades were paired so that the evolutionary path connecting the branches of the clades did not share any branches with other clades in the cladogram [52]. Hence, each comparison was phylogenetically independent. Pairings were also made between least evolutionarily distant clades and also the maximum number of pairings for maximum possible comparisons was made as in Linder [2]. Eleven pairs of temperate and tropical clades were identified. For all pairs *t*-tests with unequal variances were run. All statistical analyses were performed with Systat 13 [53] and the R programming software [50].

## Results

### Broad-Scale pattern of the relative proportions of seed oil content

In general, tropical species had significantly higher proportions of oil content than temperate species when the oil content of all species occurring in temperate and tropical conditions were considered (*t* = −5.92, df = 1988 (unequal variances), *P* <0.0001). Based on average values, temperate and tropical species had 24.6 % and 28.4 % seed oil content, respectively. The same pattern was observed when only strictly temperate or strictly tropical species were considered (t = −8.34, df = 651 (unequal variances), *P* < 0.0001), with seed oil content of 24.2 % and 32.7 % for strictly temperate and strictly tropical species.

On average, the temperate families had lower seed oil content (22.8 %) than tropical families (41.5 %). Forty two families with both temperate and tropical species were identified. Of these 42 families, 22 had a lower mean proportion of seed oil content in the temperate species when compared to the tropical species of the same family (*χ*^2^ = 173.7, df = 1, *P* < 0.001). Two-way ANOVAs for families and genera with both tropical and temperate members showed that the differences in the oil content at the family level was significant (*P* < 0.001), but was not significant at the genus level when all the families were considered (*P* = 0.191). However, a one-way ANOVA revealed that the differences in seed oil content between the strictly temperate and strictly tropical members of families which had both temperate and tropical members were significant in five families (Cyperaceae (*P* = 0.038), Zygophyllaceae (*P* = 0.012), Eleocarpaceae (*P* = 0.015), Moraceae (*P* = 0.007), Rubiaceae (*P* = 0.01), and similar patterns was observed in Fabaceae (*P* = 0.054) and Convolvulaceae (*P* = 0.051) (Table 1). In these families with the exception of Moraceae, the seed oil content of the temperate species is lesser than the tropical species. Similar trends were observed in the seed oil content between temperate and tropical members of the families Caryophyllaceae (*P* = 0.085), Proteaceae and Theaceae (*P* = 0.09) which had both temperate and tropical species where the seed oil content proportions of all the temperate species were lesser than the tropical species. In the family Combretaceae also all the temperate species had lower seed oil content than the tropical species but the differences was not significant (*P* = 0.236) (Table 1).

**Table 1:** One-way ANOVA testing whether the tropical and temperate members of families that have both tropical and temperate species differ in seed oil content

	Contrast		Mean proportion of oil (*N*)	
Family	*F*	*P*	Temperate	Tropical
Lauraceae	0.172	0.68	44.77 (28)	42.06 (14)
Liliaceae	0.331	0.574	25.32(18)	28.42(11)
Cyperaceae	5.091	0.038*	7.81 (17)	20 (1)
Menispermaceae	0.023	0.904	18.4 (1)	24.25 (2)
Papaveraceae	0.125	0.726	31.05 (37)	34.7 (1)
Ranunculaceae	1.235	0.269	25.02 (95)	36.4 (1)
Proteaceae	6.085	0.09	16.8 (4)	65 (1)
Zygophyllaceae	29.568	0.012*	10.4 (3)	35.25 (2)
Celastraceae	0.426	0.528	42.9 (8)	36.75 (4)
Eleocarpaceae	24.884	0.015*	2.9 (1)	29.4 (4)
Euphorbiaceae	1.094	0.298	33.59 (54)	37.2 (43)
Linaceae	1.193	0.355	30.25 (4)	27 (1)
Fabaceae	136.013	0.054	3.75 (2)	29 (1)
Polygalaceae	0.569	0.589	48.2 (1)	21.75 (2)
Moraceae	11.805	0.007*	34.25 (4)	14.77 (7)
Cucurbitaceae	0.123	0.729	35.64 (9)	37.865 (20)
Combretaceae	1.588	0.236	1.95 (2)	21.648 (10)
Myrtaceae	0.091	0.792	14.4 (3)	10 (1)
Onagraceae	0.282	0.598	23.864 (40)	20.4 (1)
Anacardiaceae	0.512	0.482	32.34 (20)	23.17 (3)
Rutaceae	0.09	0.770	33.24 (8)	31.27 (6)
Sapindaceae	0.691	0.414	38.07 (11)	31.75 (15)
Simourabaceae	0.665	0.431	57.3 (1)	39.19 (13)
Malvaceae	1.933	0.170	15 (27)	13.1 (28)
Caryophyllaceae	3.220	0.085	5.38 (25)	8 (1)
Capparaceae	1.762	0.209	28.57 (7)	20.93 (7)
Moringaceae	2.110	0.197	54.3 (1)	38.34 (7)
Santalaceae	0.090	0.774	47.16 (7)	54.3 (1)
Sapotaceae	0.001	0.970	35.07 (3)	34.59 (52)
Theaceae	3.516	0.090	46.3 (7)	33.12 (5)
Apocynaceae	0.302	0.6	23.2 (1)	39.25 (6)
Loganiaceae	0.271	0.694	6 (1)	24.5 (2)
Rubiaceae	10.723	0.010*	7.75 (8)	28.27 (3)
Convolvulaceae	4.915	0.051	6.78 (5)	11.57 (7)
Solanaceae	2.551	0.115	32 (47)	29 (18)
Acanthaceae	3.011	0.117	13.7 (7)	29.08 (4)
Bignoniaceae	2.287	0.169	26.8 (7)	36.97 (3)
Pedaliaceae	0.072	0.806	38.8 (3)	37 (2)
Oleaceae	0.52	0.503	15.58 (6)	24.54 (1)
Scrophulariaceae	1.264	0.266	30.4 (50)	23.37 (3)
Verbenaceae	1.129	0.313	11.19 (7)	17.87 (5)
Boraginaceae	1.604	0.208	19.87 (129)	15.5 (4)

*Significant at *P* < 0.05

Thirty one of the 34 strictly temperate families (91 %) had lower average proportions of seed oil content (23 %) than the families that were strictly tropical (43 %)(*χ*^2^ = 449.28, df = 1, *P* < 0.001,Table 2, Fig. 1). Nested ANOVA for strictly temperate and tropical families with families and latitude (temperate and tropical) as independent variables showed that there is a significant effect of latitude within families on oil content at the family level (*P* < 0.0001). A nested ANOVA test of the temperate and tropical genera of the strictly temperate and strictly tropical families when genera and latitude were treated as independent variables showed that there is a significant effect of latitude on the oil content at the genus level (*P* = 0.001). The average proportions of the oil content of the temperate genera (18.9 %) of the strictly temperate families was lower than the average proportions of oil content of the tropical genera (49.4 %) of the strictly tropical families (*χ*^2^ = 1893.9, df = 1, *P* < 0.001).

**Table 2:** One-way ANOVA testing whether the tropical and temperate members of genera that have both tropical and temperate species differ in seed oil content

	Contrast		Mean proportion of oil (*N*)	
Genus	*F*	*P*	Temperate	Tropical
*Actinodaphne*	1.167	0.475	55.9 (2)	46.73 (1)
*Cinnamomum*	2.725	0.241	30 (1)	55.2 (3)
*Lindera*	0.292	0.596	50.66 (15)	54.43 (3)
*Litsea*	0.926	0.512	57 (1)	42 (2)
*Neolitsea*	2.194	0.199	43.43 (3)	57.93 (4)
*Phoebe*	2.37	0.367	0.9 (2)	1.7 (1)
*Astelia*	0.087	0.774	26.51 (7)	28.43 (5)
*Gahnia*	4.589	0.085	9.05 (6)	20 (1)
*Glaucium*	0.047	0.849	31.4 (3)	34.7 (1)
*Clematis*	28.048	0.001*	15.33 (8)	36.4 (1)
*Elaeocarpus*	24.884	0.015*	2.9 (1)	29.03(4)
*Chrozophora*	1.355	0.364	36.57 (3)	24 (1)
*Croton*	1.459	0.314	14.9 (1)	31.3 (4)
*Euphorbia*	0.888	0.352	33.96 (35)	40 (2)
*Jatropha*	4.593	0.278	52.8 (1)	14.75 (2)
*Linum*	1.193	0.355	30.25 (4)	27 (1)
*Cucurbita*	0.187	0.681	31.2 (5)	28.17 (3)
*Terminalia*	0.490	0.523	2.10 (1)	24.46 (5)
*Oenothera*	0.257	0.616	23.2 (31)	20.4 (1)
*Zanthoxylum*	2.155	0.381	39.45 (2)	20 (1)
*Abutilon*	2.959	0.161	15.75 (2)	14.25 (4)
*Hibiscus*	0.003	0.956	13.1 (4)	13.27(15)
*Cleome*	1.014	0.371	26.3 (5)	27.2 (3)
*Moringa*	2.110	0.197	54.3 (1)	38.3 (7)
*Santalum*	0.036	0.861	49.33 (4)	54.3 (1)
*Camellia*	0.337	0.578	46.3 (7)	43.2 (3)
*Ipomoea*	1.378	0.293	9.1 (2)	12.3 (5)
*Nicotiana*	3.592	0.064	34.32 (33)	32.27 (16)
*Solanum*	34.464	0.010*	20.9 (3)	13.5 (2)
*Penstemon*	1.478	0.259	23.77 (9)	29.4 (1)
*Heliotropium*	0.957	0.4	13.4 (4)	19.6 (1)

*Significant at *P* < 0.05

At the genus level, a significant difference in the proportions of seed oil content in representatives of strictly tropical or temperate members was observed in three genera: *Clematis* (*P* = 0.001), *Elaeocarpus* (*P* = 0.015), *Solanum* (*P* = 0.01) and a similar pattern was observed in *Gahnia* (*P* = 0.085) and *Nicotiana* (*P* = 0.064). The proportions of seed oil content were greater in the strictly tropical members of the genera *Clematis* and *Elaeocarpus*, but a reverse trend was observed in the *Solanum* genus (Table 2). The differences in the seed oil content between all the temperate and tropical genera were significant when all the temperate and tropical genera across families were considered (*P* < 0.0001).

### Response of seed oil content to latitude

**Fig. 2:**
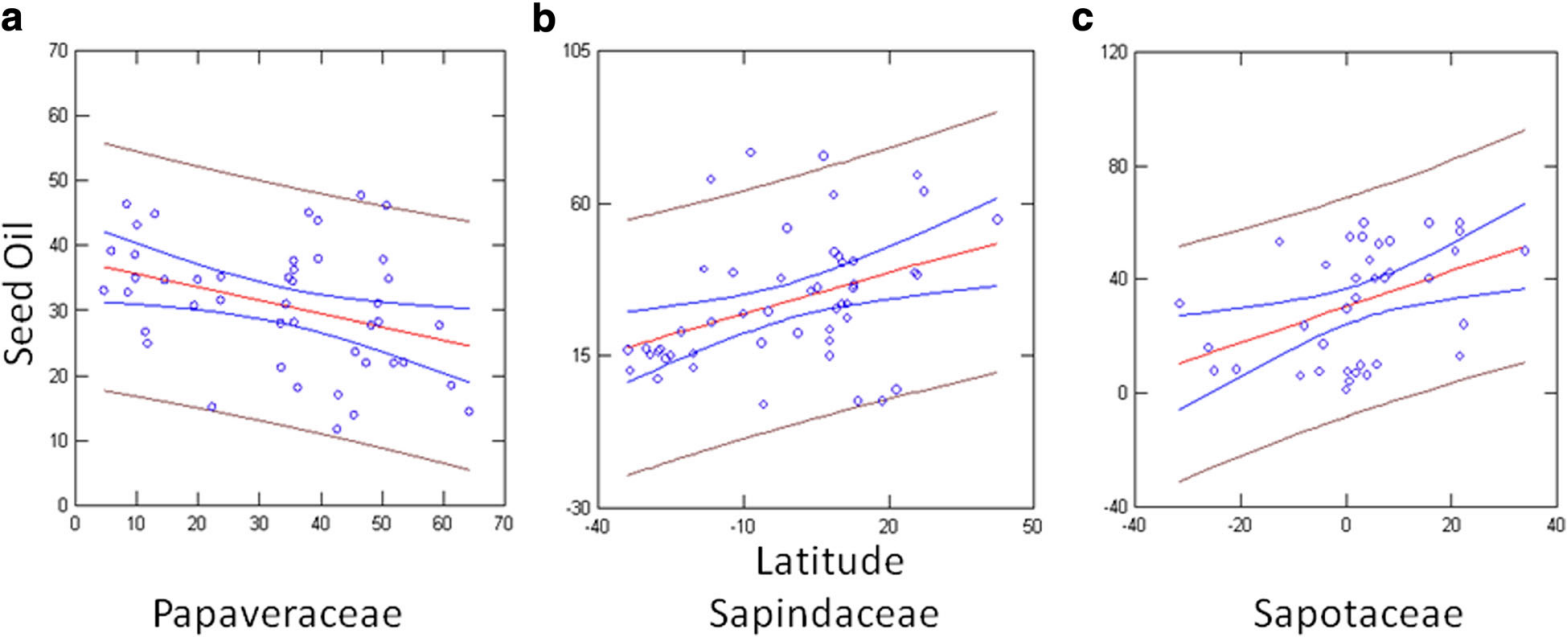
Regression of seed oil content on latitude in (**a**) Papaveraceae (**b**) Sapindaceae and (**c**) Sapotaceae

We examined the relationship between seed oil content and latitude across 168 families. The OLS regression analysis showed that the relationship between oil content and latitude was nearly significant (*P* = 0.09). A regression analysis of individual families showed that the relationship between oil content and latitude was significant in three families: Sapindaceae (*P* = 0.005), Sapotaceae (*P* = 0.007), Papavaraceae (*P* = 0.015), and was nearly significant in Solanaceae (*P* = 0.058) (Table 3, Fig. 2). The seed oil content increased with latitude in Sapindaceae and Sapotaceae and decreased with latitude in Papaveraceae.

**Table 3:** Direction, rate and significance of latitudinal changes in seed oil content in families with n > 30

Family	Direction	Slope	Significance
Anacardiaceae		0.134	0.557
Boraginaceae		−0.056	0.166
Cucurbitaceae		0.056	0.633
Cyperaceae		−0.054	0.171
Euphorbiaceae		−0.061	0.352
Lauraceae		0.181	0.363
Malvaceae		0.014	0.664
Meliaceae		−0.125	0.642
Onagraceae		0.070	0.249
Papaveraceae	Decrease	−0.204	0.015*
Ranunculaceae		−0.008	0.876
Rosaceae		−0.013	0.913
Rutaceae		0.122	0.474
Sapindaceae	Increase	0.406	0.005*
Sapotaceae	Increase	0.628	0.007*
Scrophulariaceae		0.017	0.851
Solanaceae		−0.072	0.058

*Significant at *P* < 0.05

#### Correlation analysis between seed oil content and FAs in Sapindaceae, Sapotaceae Papaveraceae

In the Sapindaceae family, a significant negative correlation between seed oil content and palmitic (16:0, *r* = −0.379, *P* =0.011) and linoleic (18:2, *r* = −0.498, *P* = 0.001) acids was observed. There was a significant positive relationship between oil content and oleic (18:1, *r* = 0.542, *P* < 0.001), arachidic (20:0, *r* = 0.540, *P* < 0.001) and eicosenoic (20:1, *r* = 0.683, *P* < 0.001) acids. In the Sapotaceae family also, a significant negative correlation was observed between seed oil content and linoleic acid (18:2, *r* = −0.613, *P* = 0.02) and a nearly significant negative correlation (*P* = 0.066) was also observed between linoleic acid and seed oil content in the Papaveraceae family. A negative correlation was observed between seed oil content and seed size in Papaveraceae but the correlation was not significant.

#### Phylogenetic analyses

Linder’s (2) method of testing for phylogenetic signals was performed. It was observed that ten of the eleven pairs of phylogenetically independent families for which oil content data was available from both latitudinal types had lower proportions of seed oil in the temperate clade, and in five pairs, the temperate clade had significantly lower proportions of oil content when compared to the tropical clade and one pair had nearly significant lower proportion of seed oil content in the temperate clade (Table 4). In no case did a tropical clade have a significant lower proportion of oil content than a temperate clade.

**Table 4:** Phylogenetically constructed *t*-tests of independent strictly temperate and strictly tropical clades (see Fig. 3)

Clades (temperate-tropical)	df	*t* value	*P* value
Xanthorrhoeaceae-Arecaceae	3	2.53	0.043*
Paeoniaceae-Calophyllaceae	4	3.31	0.015*
Grossulariaceae-Chrysobalanceae	20	−6.79	<0.0001*
Fagaceae-Vochysiaceae	9	16.42	<0.0001*
Nyssaceae-Opiliaceae	1	8.39	0.04*
Betulaceae-Achariaceae	2	0.9	0.23
Saxifragaceae-Achariaceae	1	0.79	0.29
Daphniphyllaceae-Clusiaceae	4	1.14	0.16
Calycanthaceae-Hernandiaceae	2	0.3	0.4
Xanthorrhoeaceae-Orchidaceae	1	0.61	0.33
Lardizabalaceae-Arecaceae	5	−1.69	0.08

*Significant at *P* < 0.05

**Fig. 3:**
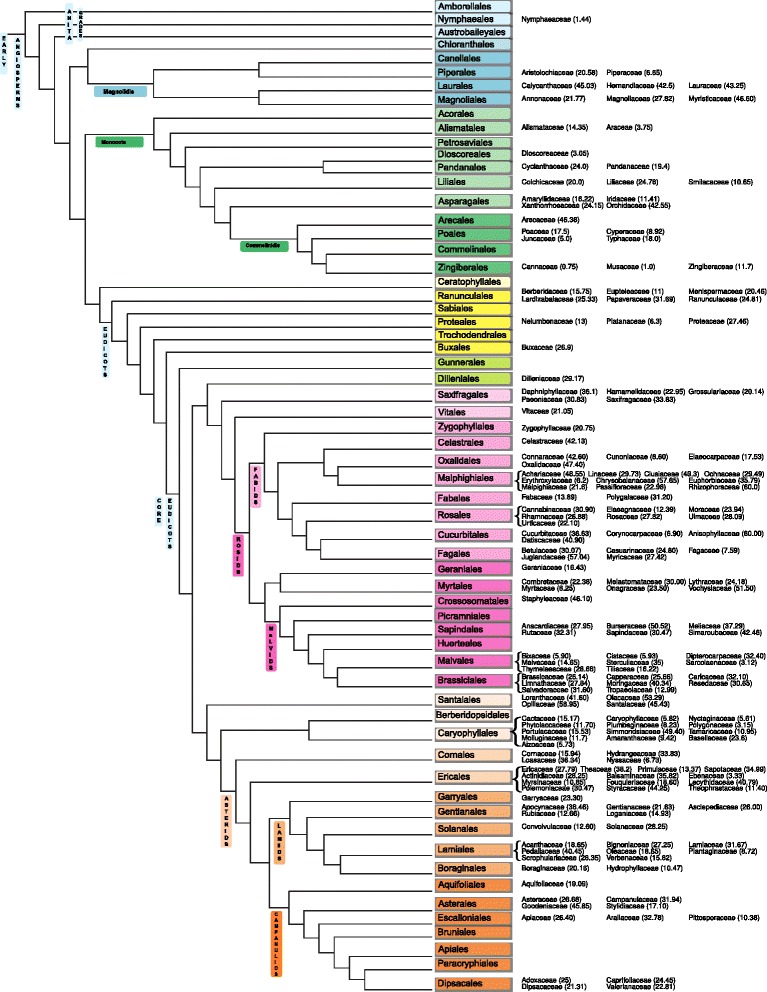
Seed oil content in 168 angiosperm families mapped onto the angiosperm phylogeny tree adapted from Stevens PF (2001 onwards)

Furthermore, consistently high (≥20 %) or low (<20 %) seed oil content proportions [54] were observed in the families of eight (Laurales, Magnoliales, Alismatales, Poales, Zingiberales, Saxifragales, Sapindales and Santalales) of the 62 Angiosperm clades at the order level (Fig. 3). In the Magnoliids clade, the clades Laurales (42.5 to 45 %) and Magnoliales (21.8 to 46.6 %) evolved independently to have high oil content while in the Monocot clade, the members of the clade Alismatales (3.6 to 14.4 %), Poales (5-18 %) and Zingiberales (0.8 to 11.7 %) which belong to the Commelinids clade within Monocots evolved independently to have low oil content (Fig. 3). In the core Eudicots clade, members of the Saxifragales (20.1 to 36.1 %), Sapindales (28 to 50.5 %) clades evolved independently to have high oil content (Fig. 3) and in the Asterids clade, the members of the clade Santalales evolved independently to have high seed oil content (41.6 to 59 %) as they did not share a common evolutionary path.

In addition, in the core Eudicots clade, the Ranunculales clade had two groupings where low oil content (≤15 %) was observed in two families and high oil content (20.5 to 31.7 %) in three families (Additional file 1: Table S1). In the Fabids group under Rosids, it has been observed that the clades of the orders Malphighiales, Rosales, Cucurbitales and Fagales have higher oil content with the exception of one family in each of these clades. In the Malphigiales clade, 9 families had high oil content (21.6 to 60 %) with the exception of Erythroxylaceae (6.2 %). In the clade Rosales, the oil content of six families had high oil content (22.1 to 30 %) with the exception of Eleagnaceae (12.4 %). The members of the Cucurbitales clade had high oil content (36.1 to 60 %) with the exception of Corynocarpaceae (6.9 %). The members of the Fagales clade, had high oil content (24.8 to 52.4 %) with the exception of Fagaceae (7.6 %). Within the Malvids clade, the order Myrtales had high oil content (22.4 to 51.5 %) with the exception of Myrtaceae (8.3 %). The members of the order Malvales evolved to have lower oil content (3.1 to 15 %) with the exception of the families Thymelaeaceae (28.7 %) and Dipterocarpaceae (32.4 %). However, the families Erythroxylaceae, Eleagnaceae and Corynocarpaceae and Dipterocarpaceae had only one representative member. In the Brassicales clade, the member families had high oil content (25.7 to 40.3 %) with the exception of Tropaeolaceae (13 %) (Table 3, Fig. 3).

In the Asterids clade, the members of the clade Caryophyllales evolved to produce low oil content (3 to 17 %) with the exception of Simmondsiaceae (49.4 %). In the Ericales clade, there were two distinct grouping with lower oil content (5 to 18.6 %) in five families and high oil content (27.8 to 44.3 %) in eight families. In the Boraginales clade, the oil content of the members was low (10.5 to 20.1 %) (Fig. 3).

Studying 168 families revealed that families belonging to clades of eight orders in the Angiosperm phylogeny [50] evolved to have low or high seed oil content. Of these clades of individual families for which seed oil content data of at least 2 species of the family was available, 34 clades at the family level were strictly temperate and 12 clades were strictly tropical (Additional file 1: Table S1, Fig. 3). An additional four clades (Myristaceae, Ochnaceae, Moringaceae, Lecythidaceae) were primarily tropical with the exception of one species, and nine clades were primarily temperate (Amaryllidaceae, Eleagnaceae, Geraniaceae, Plumbaginaceae, Cornaceae, Polemoniaceae, Garryaceae, Aquifoliaceae, Pittosporaceae) with the exception of one species. A total of 111 clades at the family level have species occurring both in the temperate and tropical regions and also the same species occurring in both the biogeographical regions.

## Discussion

### Evidence of differences in oil content in different biogeographical regions

A significant difference in the seed oil content of temperate and tropical plants was observed in this study. Characteristics of seeds, especially the stored energy in seed oils and the nature of seed oil is crucial for successful germination and reproduction. Oils are found in at least 83 (30 %) of the plant families listed by Engler and Prantl [55], and the majority of these 83 families (up to 48.1 %) are mainly tropical or subtropical and up to 18 % are mainly temperate in habitat [55]. Hence, oils are found more frequently in plants in tropical and subtropical regions than in temperate regions. The adaptive evolution of seed oil composition in temperate and tropical regions has been shown in previous studies [2]. Furthermore, high temperature is known to have a positive influence on seed oil content [56]. Since the oilseed plants are more frequent in tropical and subtropical regions, observed increase in oil content in subtropical and tropical herbs when compared to the temperate herbs [6] possibly helps in adaptation as greater packing of species and greater competitive interactions in the tropics may select for greater energy level in the seeds [5]. This pattern of increasing seed oil content from temperate to subtropical to tropical herbs was also observed in Brassicaceae (Sanyal et al. unpubl. data). Significant differences in seed oil content within herbaceous species across different biomes in the family Brassicaceae was observed. A shift in temperature in the different biomes is accompanied by an increase in content of a more efficient storage product like oil. In the tropics, *K*-selection will be favoured over *r*-selection which will lead to selection of seeds rich in energy reserves [6].

In majority of the families (52.4 %) with both temperate and tropical species, a significant difference in seed oil content between temperate and tropical species was observed where the tropical species have higher proportions of seed oil content. Several families (35.7 %) had ≤ 4.79 % difference in seed oil content between the temperate and tropical species and the variation may not be enough for selection to act on seed oil content in these families or alternatively suggests the presence of purifying selection on seed oil content in these families. In addition, in families like Lauraceae where several species are adapted to swamps, Liliaceae where both asexual and sexual reproduction is present, Menispermaceae where several species lack endosperm, seed oil content may not be under selection and may not show the pattern observed in the seed oil content rich families (Table 1) while other seed characteristics, like the size and architecture will be under selection. In addition, in families like Alismataceae, the absence of this pattern could be due to the presence of many aquatic species where selection is unlikely to act on seed oil content but other seed traits like size and structure.

The results in this study show that there is a significant difference in the seed oil content between the temperate and tropical species. The results are significant both in non-phylogenetic and phylogenetic contexts based on the assumption that selection will erase any phylogenetic signal.

The proportions of seed oil content in strictly temperate species (24.2 %) were significantly lower than tropical species (32.7 %). Previous studies have shown that germination temperature selects for a greater proportion of unsaturated fatty acids at higher latitude [2]. It is possible that in the tropics there is more competition and a greater proportion of seed oil content with greater proportions of saturated FAs gives the plants a competitive advantage helping them to germinate quickly and grow faster especially if they are understorey plants in crowded tropical regions. The greater the seed oil content and saturated FAs, the greater the energy reserve, the greater will be the probability of seedling establishment during low illumination or high competition [57].

### Evidence of a latitudinal cline in individual families

The relative proportion of seed oil content has been observed to change with latitude in this study. The study of three families (Sapindaceae, Sapotaceae and Papaveraceae) indicates that a selective pressure correlated with latitude acts on the proportions of seed oil content in Angiosperms. It was observed that in the families Sapindaceae and Sapotaceae there was a significant increase in the proportions of seed oil content with increasing latitude. This pattern was also observed in *Arabidopsis thaliana* in Brassicaceae (Sanyal et al. unpubl. data). Studies have also shown that small seeds have higher oil content which are richer in polyunsaturated fatty acids [58]. The pattern observed in *A. thaliana*, Sapotaceae and Sapindaceae species suggests that selection could be acting on multiple seed traits: seed size, seed oil content and seed oil composition to facilitate faster growth and reproduction at higher latitudes or lower temperatures. A reverse trend where the oil content decreased with increasing latitude was observed in Papaveraceae indicating that high temperature favours increased oil content which helps the plant to germinate quickly and compete better in tropical regions as there is more species crowding in tropical regions. As observed by Bretagnolle et al. [58] a negative correlation between seed oil content and seed size was also observed in Papaveraceae. A larger sample size could provide a better understanding of this relationship between seed size, seed oil content and composition and latitude. This trend of an inverse relationship between seed oil content and latitude was also observed in the broad-scale study across all the species of all the families in Angiosperms which showed that the relationship between latitude and seed oil content was nearly significant (*P* =0.092). This pattern was also observed in Solanaceae and was nearly significant (*P* = 0.058). Seeds of Sapotaceae, Papaveraceae and Sapindaceae species are rich in oil and show a great variation in seed size [59–61], seed oil content and composition [9] suggesting that seed size and seed oil content and composition could be under selection. However, since the occurrence data was extracted from GBIF; there is a possibility that the occurrence data reported from all over the world is incomplete and inconsistent due to differences in sampling efforts across the world and more data might reveal the true relationship across families. Additional data on seed size, seed oil content and oil composition will give us an understanding of how these traits contribute towards plant adaptation.

Furthermore, correlation analyses of seed oil content and composition (constituent FAs) in Sapindaceae revealed that there is a significant negative correlation between seed oil content and palmitic and linoleic acids and significant positive correlations with oleic, arachidic and eicosenoic acids. A regression analysis showed that the proportions of unsaturated FAs oleic and eicosenoic acids increased significantly (*P* < 0.05) with latitude as expected; thus helping in early germination and adaptation of the plants at different latitudes and temperatures as predicted in the adaptive theory [2]. In *A. thaliana*, the proportions of saturated palmitic acid was also inversely proportional to latitude while the proportions of unsaturated oleic, linoleic and eicosenoic acids increased with latitude as expected. A significant negative correlation between seed oil content and palmitic acid (16:0) and significant positive relationships between oil content and stearic, oleic, linolenic and eicosenoic acids were observed in *A. thaliana* (Sanyal et al. unpubl. data). Furthermore, *A. thaliana* has small seeds and has a greater oil content with greater proportions of polyunsaturated FAs. This suggests that selection could be acting on multiple seed traits like seed oil content and composition and size facilitating the successful growth and reproduction of the plant in different biomes.

Herbivory and photoperiod are the other important non-temperature related factors which could covary with latitude. It has been suggested that herbivory increases at lower latitudes [62–64] and herbivores prefer seeds with higher oil content as it provides more energy. It has been observed that wild birds have a preference for black-oil sunflower varieties with the highest oil content [65]. Photoperiod and temperature had an effect on the reproductive period and seed oil content in soybean [66]. Photoperiod at higher latitudes is longer in the summer and shorter during the winter. The plants subjected to a long photoperiod had higher oil content than those subjected to shorter photoperiod conditions [67]. Furthermore, the level of illumination has a significant effect on seed oil content. In temperate herbs, it has been observed that the mean oil content is inversely proportional to illumination and seed oil content increases from 16.59 % to 25.43 % to 27.91 % as level of illumination decreases in those inhabiting open habitats, woodland borders, and shaded habitats and woodlands [6].

### Phylogenetic signals in seed oil content

Pairwise comparisons of eleven pairs of strictly temperate and strictly tropical families showed that five pairs had significantly greater proportions of seed oil in the tropical families and in all cases lesser seed oil content proportions were in the temperate family. So, the results are robust whether the analyses are taken within a phylogenetic context or on the assumption that selection has erased the phylogenetic history. The broad-scale pattern results make it clear that the pattern holds broadly within angiosperms. Eight of 62 clades at the order level (Laurales, Magnoliales, Alismatales, Poales, Zingiberales, Saxifragales, Sapindales and Santalales) have shown to have very similar oil content (Fig. 3). All the families of the clades Alismatales (≤14.4 %), Zingiberales (≤11.7 %), and Poales (≤18 %) have evolved to have low seed oil content while all the families within Laurales (≥42.5 %), Magnoliales (≥27.8 %), Saxifragales (≥20.1 %), Sapindales (≥28 %) and Santalales (≥41.6 %) have evolved to have high seed oil content (Fig. 3). Alismatales also comprise of aquatic species which could influence the oil content as seed oil content may not be under selection. In some clades like Ranunculales, Malphighiales, Rosales, Cucurbitales, Fagales, Myrtales, Brassicales, and Calophyllales, all the families have an average low or high seed oil content with the exception of one (Fig. 3). Low seed oil content was also observed within Malvales with the exception of two families (Dipterocarpaceae, Thymelaeaceae) which had high seed oil content (Fig. 3). In addition, there are some taxonomic groups where the proportions of seed oil in tropical and temperate members of the families are nearly identical (Tables 1, 2 and 4) possibly due to phylogenetic constraints or other factors. Additional studies will be needed to determine why these anomalies exist and whether these exceptions could be explained within the framework of the adaptive theory like delayed germination at warmer temperatures or lack of competition during germination and establishment. However, this signal has been observed in few clades scattered across the phylogeny suggesting that seed oil content is an adaptive trait which as expected may have erased any phylogenetic signals in majority of the clades.

Furthermore, gene phylogenies of several seed oil content genes would help us understand the genetic basis of the adaptive nature of seed oil content in Angiosperms. A *FAE1* gene phylogeny of Brassicaceae suggested that purifying selection is the major evolutionary force acting on the gene which is consistent with similar findings in another fatty acid elongase gene *EVOVL5* responsible for encoding an enzyme involved in the biosynthesis of long-chain PUFAs in fishes [39]. This suggests that since *FAE1* is involved in TAG biosynthesis; focus on the sequence variation and phylogeny of genes responsible for seed oil content will be useful in understanding the evolutionary pattern of these genes in Angiosperms. Hence, several seed oil content genes should be used for generating phylogenies and a comparison of the tree topologies may reveal meaningful and robust patterns of adaptive evolution and/or phylogenetic signals in oil content in Angiosperms.

## Conclusion

This study shows that the temperate species have lower proportions of oil content than the tropical species across 168 angiosperm families both in the phylogenetic and non-phylogenetic context. A significant relationship between seed oil content and latitude was observed in three families: Sapindaceae, Sapotaceae and Papaveraceae suggesting the possible role of seed oil content in the adaptive evolution of plants. The study also reveals the relationship between constituent fatty acids in seed oil and latitude in Sapindaceae, Sapotaceae and Papaveraceae and also the correlation between seed oil content and composition and seed size indicating that selection could be acting on multiple seed traits, seed oil content and seed oil composition and size to aid plant adaptation. Finally, both non-phylogenetic and phylogenetic approaches in the study helped us understand the adaptive nature of seed oil content in Angiosperms in different biogeographical regions. Understanding the evolutionary patterns of seed oil distribution will help understand their role in plant adaptation and will also help in the breeding of oilseed crops for human consumption and also for other industrial uses.

## Additional file

Additional file 1: Table S1. Species seed oil content across families. (DOCX 19 kb)
