# Supplementary material for: Adaptive evolution of seed oil content in angiosperms: accounting for the global patterns of seed oils
Source: BMC Evol Biol. 2016 Sep 9;16(1):187. doi: 10.1186/s12862-016-0752-7 (PMC5017040; doi:10.1186/s12862-016-0752-7)
Supplement: Additional file 1: Table S1. — Species seed oil content across families. (DOCX 19 kb) [file 12862_2016_752_MOESM1_ESM.docx]

Table S1:Species seed oil content across families

| Family | Genera | Species | Mean Oil content (%) |
| --- | --- | --- | --- |
| Nymphaeaceae | 3 | 5 | 1.44 |
| Aristolochiaceae | 1 | 4 | 20.58 |
| Piperaceae | 2 | 2 | 6.65 |
| Calycanthaceae* | 2 | 3 | 45.03 |
| Hernandiaceae** | 1 | 2 | 42.5 |
| Lauraceae | 16 | 65 | 43.25 |
| Annonaceae | 8 | 13 | 21.77 |
| Magnoliaceae | 2 | 6 | 27.82 |
| Myristicaceae | 12 | 28 | 46.60 |
| Alismataceae | 2 | 2 | 14.35 |
| Araceae | 2 | 2 | 3.75 |
| Dioscoreaceae | 1 | 2 | 3.05 |
| Cyclanthaceae | 1 | 1 | 24 |
| Pandanaceae** | 1 | 1 | 19.4 |
| Colchicaceae* | 1 | 1 | 20 |
| Liliaceae | 10 | 23 | 24.76 |
| Smilacaceae | 1 | 2 | 10.65 |
| Amaryllidaceae | 4 | 5 | 16.22 |
| Xanthorrhoeaceae* | 1 | 2 | 24.15 |
| Iridaceae | 5 | 11 | 11.41 |
| Orchidaceae** | 1 | 2 | 42.55 |
| Arecaceae** | 4 | 4 | 46.38 |
| Cyperaceae | 12 | 32 | 8.92 |
| Juncaceae | 1 | 1 | 5 |
| Poaceae | 1 | 1 | 17.5 |
| Typhaceae | 1 | 1 | 18 |
| Cannaceae | 1 | 2 | 0.75 |
| Musaceae** | 1 | 1 | 1 |
| Zingiberaceae | 4 | 6 | 11.7 |
| Berberidaceae | 5 | 6 | 15.75 |
| Eupteleaceae* | 1 | 1 | 11 |
| Lardizabalaceae* | 2 | 3 | 25.33 |
| Menispermaceae | 4 | 5 | 20.46 |
| Papaveraceae | 15 | 48 | 31.69 |
| Ranunculaceae | 23 | 105 | 24.81 |
| Nelumbonaceae* | 1 | 1 | 13 |
| Platanaceae* | 1 | 1 | 6.3 |
| Proteaceae | 5 | 8 | 27.46 |
| Buxaceae | 2 | 3 | 26.9 |
| Dilleniaceae | 3 | 3 | 29.17 |
| Daphniphyllaceae* | 1 | 2 | 36.1 |
| Grossulariaceae* | 1 | 8 | 20.14 |
| Hamamelidaceae | 2 | 2 | 22.95 |
| Paeoniaceae* | 1 | 3 | 30.83 |
| Saxifragaceae* | 4 | 9 | 33.83 |
| Vitaceae | 3 | 7 | 21.05 |
| Zygophyllaceae | 7 | 11 | 20.75 |
| Celastraceae | 7 | 17 | 42.13 |
| Connaraceae | 2 | 2 | 42.60 |
| Cunoniaceae* | 1 | 1 | 8.60 |
| Elaeocarpaceae | 1 | 7 | 17.53 |
| Oxalidaceae | 2 | 4 | 47.40 |
| Achariaceae** | 1 | 2 | 46.55 |
| Clusiaceae** | 3 | 7 | 49.30 |
| Chrysobalanaceae** | 7 | 19 | 57.65 |
| Erythroxylaceae | 1 | 1 | 6.20 |
| Euphorbiaceae | 44 | 161 | 35.79 |
| Linaceae | 1 | 9 | 29.73 |
| Malpighiaceae | 5 | 5 | 21.60 |
|  |  |  |  |
| Family | Genera | Species | Mean Oil content (%) |
| Passifloraceae | 1 | 5 | 22.98 |
| Ochnaceae | 4 | 7 | 29.49 |
| Rhizophoraceae | 1 | 1 | 60.00 |
| Fabaceae | 10 | 10 | 13.89 |
| Polygalaceae | 5 | 5 | 31.20 |
| Cannabinaceae | 1 | 2 | 30.90 |
| Elaeagnaceae | 2 | 10 | 12.39 |
| Moraceae | 10 | 22 | 23.94 |
| Rhamnaceae | 11 | 24 | 26.88 |
| Rosaceae | 30 | 89 | 27.82 |
| Ulmaceae | 6 | 12 | 28.09 |
| Urticaceae | 4 | 7 | 22.10 |
| Anisophyllaceae** | 1 | 1 | 60.00 |
| Corynocarpaceae* | 1 | 1 | 6.90 |
| Cucurbitaceae | 27 | 77 | 36.63 |
| Datiscaceae* | 1 | 1 | 40.90 |
| Betulaceae* | 3 | 7 | 30.07 |
| Casuarinaceae | 1 | 3 | 24.80 |
| Fagaceae* | 3 | 28 | 7.59 |
| Juglandaceae | 3 | 11 | 57.04 |
| Myricaceae | 1 | 6 | 27.42 |
| Geraniaceae | 2 | 8 | 16.43 |
| Combretaceae | 5 | 16 | 22.38 |
| Melastomataceae | 1 | 1 | 30.00 |
| Lythraceae | 5 | 19 | 24.18 |
| Myrtaceae | 7 | 11 | 8.25 |
| Onagraceae | 8 | 54 | 23.50 |
| Vochysiaceae** | 2 | 2 | 51.50 |
| Staphyleaceae* | 1 | 1 | 46.10 |
| Anacardiaceae | 16 | 38 | 27.95 |
| Burseraceae | 6 | 17 | 50.52 |
| Meliaceae | 17 | 39 | 37.29 |
| Rutaceae | 22 | 51 | 32.31 |
| Sapindaceae | 22 | 57 | 30.47 |
| Simaroubaceae | 11 | 18 | 42.46 |
| Bixaceae | 1 | 1 | 5.90 |
| Cistaceae* | 3 | 11 | 5.93 |
| Dipterocarpaceae** | 1 | 1 | 32.40 |
| Malvaceae | 24 | 126 | 14.65 |
| Sterculiaceae | 1 | 1 | 35 |
| Sarcolaenaceae | 8 | 18 | 3.12 |
| Thymelaeaceae | 2 | 5 | 28.66 |
| Tiliaceae | 8 | 17 | 16.22 |
| Brassicaceae | 89 | 230 | 26.14 |
| Capparaceae | 8 | 23 | 25.66 |
| Caricaceae | 1 | 1 | 32.10 |
| Limnathaceae* | 1 | 14 | 27.84 |
| Moringaceae | 1 | 8 | 40.34 |
| Resedaceae | 1 | 3 | 30.65 |
| Salvadoraceae | 2 | 3 | 31.60 |
| Tropaeolaceae | 3 | 4 | 12.99 |
| Loranthaceae | 1 | 1 | 41.60 |
| Olacaceae | 7 | 12 | 53.29 |
| Opiliaceae** | 1 | 2 | 58.95 |
| Santalaceae | 4 | 9 | 45.43 |
| Aizoaceae* | 2 | 3 | 5.73 |
| Amaranthaceae | 7 | 23 | 9.42 |
| Basellaceae | 1 | 1 | 23.6 |
| Cactaceae | 3 | 6 | 15.17 |
| Caryophyllaceae | 11 | 29 | 5.82 |
|  |  |  |  |
| Family | Genera | Species | Mean Oil content (%) |
| Nyctaginaceae | 2 | 3 | 5.61 |
| Phytolaccaceae | 1 | 1 | 11.70 |
| Plumbaginaceae | 3 | 4 | 8.23 |
| Polygonaceae | 6 | 28 | 3.15 |
| Portulacaceae | 1 | 2 | 15.53 |
| Simmondsiaceae* | 1 | 1 | 49.40 |
| Tamaricaceae | 1 | 1 | 10.95 |
| Cornaceae | 4 | 9 | 15.94 |
| Hydrangeaceae* | 3 | 3 | 33.83 |
| Loasaceae* | 2 | 5 | 36.34 |
| Nyssaceae* | 1 | 2 | 6.73 |
| Actinidiaceae* | 1 | 2 | 28.25 |
| Balsaminaceae | 2 | 9 | 35.82 |
| Ebenaceae | 1 | 5 | 3.33 |
| Ericaceae | 3 | 7 | 27.79 |
| Fouquieriaceae* | 1 | 1 | 18.60 |
| Lecythidaceae | 4 | 8 | 40.79 |
| Myrsinaceae | 2 | 2 | 10.85 |
| Polemoniaceae | 4 | 6 | 30.47 |
| Primulaceae* | 3 | 13 | 13.37 |
| Sapotaceae | 24 | 46 | 34.99 |
| Styracaceae* | 1 | 2 | 44.25 |
| Theaceae | 4 | 19 | 38.20 |
| Theophrastaceae | 1 | 1 | 11.40 |
| Garryaceae | 1 | 4 | 23.30 |
| Apocynaceae | 17 | 23 | 36.46 |
| Asclepediaceae | 1 | 1 | 26.00 |
| Gentianaceae* | 2 | 4 | 21.63 |
| Loganiaceae | 2 | 4 | 14.93 |
| Rubiaceae | 17 | 26 | 12.66 |
| Convolvulaceae | 6 | 36 | 12.60 |
| Solanaceae | 17 | 116 | 28.25 |
| Acanthaceae | 12 | 15 | 18.65 |
| Bignoniaceae | 16 | 16 | 27.25 |
| Lamiaceae | 4 | 4 | 31.67 |
| Pedaliaceae | 5 | 11 | 40.45 |
| Oleaceae | 8 | 11 | 18.65 |
| Plantaginaceae* | 1 | 19 | 8.72 |
| Scrophulariaceae | 21 | 67 | 28.35 |
| Verbenaceae | 15 | 19 | 15.82 |
| Boraginaceae | 43 | 153 | 20.16 |
| Hydrophyllaceae* | 4 | 9 | 10.47 |
| Aquifoliaceae | 1 | 7 | 19.09 |
| Asteraceae | 10 | 12 | 26.68 |
| Campanulaceae | 6 | 9 | 31.94 |
| Goodeniaceae | 2 | 3 | 45.85 |
| Stylidiaceae | 1 | 1 | 17.10 |
| Apiaceae | 1 | 1 | 26.40 |
| Araliaceae | 5 | 12 | 32.78 |
| Pittosporaceae | 1 | 4 | 10.38 |
| Adoxaceae | 1 | 1 | 25 |
| Caprifoliaceae* | 4 | 17 | 24.45 |
| Dipsacaceae* | 4 | 13 | 21.31 |
| Valerianaceae*  Molluginaceae | 4  2 | 7  2 | 22.81  11.7 |

*Strictly temperate families

**Strictly tropical families
